# Enhancing Physical and Cognitive Efficiency in Elderly Individuals at Risk for Dementia Using Whole-Body Electrostimulation: A Randomized Controlled Trial

**DOI:** 10.3390/jfmk9040246

**Published:** 2024-11-23

**Authors:** Marco Centorbi, Giulia Di Martino, Carlo Della Valle, Andrea Buonsenso, Giuseppe Calcagno, Giovanni Fiorilli, Alessandra di Cagno

**Affiliations:** 1 Department of Medicine and Health Sciences, University of Molise, 86100 Campobasso, Italy; marco.centorbi@hotmail.it (M.C.); giulia.dimartino21@gmail.com (G.D.M.); or carlo.dellavalle@univr.it (C.D.V.); fiorilli@unimol.it (G.F.); 2 Department of Neurosciences, Biomedicine and Movement, University of Verona, 37314 Verona, Italy; 3 Department of Movement, Human and Health Sciences, University of Rome “Foro Italico”, 00135 Rome, Italy; alessandra.dicagno@uniroma4.it; 4 Department of Human Sciences, Guglielmo Marconi University, 00193 Rome, Italy

**Keywords:** WB-electromyostimulation, EMS, electrical muscle stimulation, muscle strength, neurodegenerative disease, cardiovascular training

## Abstract

**Objective**: The aim of this randomized controlled trial (RCT) was to assess the impact of a 12-week intervention of two 20-min sessions per week, combining aerobic exercise with whole-body electromyostimulation (WB-EMS), on physical and cognitive performance in the elderly. **Methods**: A total of 61 participants (age = 71 ± 5.64 years), healthy or at risk for dementia, were randomly assigned to an experimental training group (ETG, n = 33) and a control group (CON, n = 28). Participants underwent 20-min aerobic training sessions, with intensity increasing from 60% to 80% of heart rate reserve (HRR), with and without continuous WB-EMS stimulation (35 Hz, 350 μs). **Results**: Significant time/effects for both the ETG and CON were found in the physical performance tests, with significant time*group interactions favoring the ETG for the arm curl test (*p* < 0.001) and the sit-to-stand test, with significant differences between groups (*p* = 0.001), as well as for the hand grip test (*p* < 0.001) and the 6-min walking test (*p* < 0.001), with significant time*group interactions (*p* = 0.003). Both groups improved their performance on the soda pop test (*p* < 0.001). ETG outperformed CON in memory performance (PROSA, *p* = 0.046; RAVLT immediate recall, *p* < 0.001) and on selective attention and visuospatial processing (attention matrices, *p* = 0.014). Some cognitive tests showed no significant improvement, likely due to the short intervention period for cognitive function (MMSE, *p* = 0.628; TMT, *p* = 0.698; Stroop error, *p* = 0.188) or memory performance (PROSA, *p* = 0.338). **Conclusion**: The absence of decline suggests a protective effect of physical activity. WB-EMS, combined with aerobic training, enhances the benefits of physical activity and helps counteract cognitive decline in older adults.

## 1. Introduction

Population aging is one of the most significant demographic trends of the 21st century, marked by a steady increase in the proportion of elderly individuals (over 65 years) within the global population, particularly in developed countries. The key factors driving this phenomenon are increased life expectancy and declining birth rates [1]. These trends underscore significant challenges for healthcare and social systems, emphasizing the need for public policies that address the growing demand for resources and services required by the aging population, increasing the costs of the social and healthcare system in order to ensure healthy aging [2]. The increased lifespan is also associated with a decline in functional ability and a rise in disability [3]. The reduction in the working-age population could slow economic growth and shift the demand for goods and services such as healthcare services and assistive technologies. Therefore, it is essential to develop and promote strategies that enhance the quality of life for older adults and reduce the healthcare burden [4]. Physical activity (PA) has proven to be one of the most effective approaches in this strategy. Regular physical exercise, both aerobic and resistance-based, not only helps maintain physical health but also has a significant positive impact on cognitive function [5]. Considering that the use of neuroprotective drugs may have side effects and represents a significant expense for public healthcare, PA could be a good strategy to prevent or delay the onset of the consequences of aging, such as a decline in cognitive and physical function [6]. A meta-analysis by Smith et al. [7] (2010) found that PA interventions significantly improved global cognitive function, with particularly strong effects on executive functions and memory. PA, particularly aerobic exercise programs, have a direct impact on brain plasticity and neurogenesis by promoting the production of neurotrophic factors like brain-derived neurotrophic factor (BDNF), which helps prevent cognitive decline. Additionally, aerobic training reduces cardiovascular risk factors, lowers stress, and improves sleep quality, all of which enhances overall cognitive function, particularly executive functions, even in older adults with mild cognitive impairment (MCI) [8].

To ensure the proper implementation of PA protocols, it is essential to motivate participants, as the benefits are only guaranteed through adherence to the protocols [9]. In this regard, technology has played a significant role in facilitating PA and promoting adherence among the elderly [10].

Whole-body electromyostimulation (WB-EMS) training is an emerging technology effective in improving both physical efficiency and cognitive functions. WB-EMS provides electrical stimulation to the entire body, activating up to 8–10 muscle groups simultaneously. This enables the exercise of different kinetic chains while performing functional movements during the stimulation. Additionally, WB-EMS training has been shown to modulate serum proteins such as BDNF and nerve growth factor (NGF) in Parkinson’s disease patients [11]. Individuals undergoing WB-EMS may be more motivated to continue by seeing rapid improvements [12]. WB-EMS is also less exhausting compared to traditional PA, well-tolerated, and promotes exercise adherence among sedentary individuals. Therefore, WB-EMS, combined with physical exercise, is a promising modality for those who cannot or do not wish to engage in conventional PA [13].

This study aimed to assess the effect of 12 weeks of WB-EMS, combined with aerobic training, on physical and cognitive performance in older adults, whether healthy or at risk for dementia, compared to the same training protocol without WB-EMS.

## 2. Materials and Methods

### 2.1. Study Design

The study is a randomized controlled trial (RCT) designed to determine the effects of 20-min exercise sessions, employing a WB-EMS intervention combined with aerobic exercise, performed on a treadmill and rowing machine, on physical and cognitive efficiency in elderly individuals. The study is fully registered under www.clinicaltrials.gov (NCT06669598) (accessed on 20 October 2024).

### 2.2. Participants

A total of 66 participants (age = 71 ± 5.64 years) were recruited from the Center for Research and Training in Aging Medicine of Molise (Ce.R.M.I.) and were randomly assigned to the experimental training group (ETG) (n = 33; age = 70 ± 6.52 years) or to a control group (CON) (n = 33; age = 72.42 ± 4.76 years). After the baseline assessment, each of the 61 enrolled participants was given a unique number. A random number list was generated using online software (https://www.random.org/sequences/ (accessed on 20 January 2024), Dublin, Ireland) containing numbers 1 to 61, with no repetitions. This list was used to randomly order and allocate the participants into the respective groups. However, five participants in the CON group dropped out during the experimental procedure due to personal reasons. Therefore, the final number of participants enrolled in the CON group was twenty-eight (71.87 ± 4.56).

The characteristics of the participants are shown in Table 1.

The sample size was calculated using G*Power (version 3.1.9.7; written by Franz Faul, University of Kiel, Kiel, Germany). The following design specifications were considered: test family = F tests; statistical test = repeated measures of analysis of variance (ANOVA) between factors; α = 0.05; (1 − β) = 0.95; effect size f = 0.5; number of groups = 2; number of measurements = 2. The sample size estimation indicated that 42 participants were required, with a critical F value of 4.085.

The inclusion criteria were as follows: (1) age from 55 to 85 years; (2) no simultaneous participation in any type of supervised physical activity; (3) sedentary lifestyle. The following exclusion criteria were also applied: a Mini-Mental State Examination (MMSE) score of less than 24; inability to walk for 6 min without assistance; the presence of a medical condition influencing the cognitive and/or motor functions; the presence of any counterindication for the utilization of EMS, such as cardiovascular diseases, stents, cardiac pacemakers, and diabetes mellitus, verified by medical certification.

All participants were informed about the study purpose and procedures, and each provided written informed consent. The study was designed and conducted in accordance with the Declaration of Helsinki and received approval from the Ethics Committee of the Azienda Sanitaria Regionale Molise-ASREM (11487/2020).

### 2.3. Experimental Procedures

A familiarization session was conducted one week before the intervention to allow participants to learn the correct exercise techniques and to determine the optimal stimulation intensity for each muscle group for each participant. Each muscle group was stimulated intermittently to ascertain the maximum tolerable stimulus for each participant. The description of WB-EMS device is provided in supplementary material. The final stimulation value delivered by the EMS was recorded [14].

The ETG group underwent two 20-min training sessions per week, with 72 h of rest between sessions. Each session included aerobic activities such as rowing and treadmill exercises, combined with supervised WB-EMS stimulation. This stimulation utilized a rectangular waveform at 35 Hz, 350 μs, with continuous pulse duration. The intensity of this training modality was set based on heart rate reserve (HRR), starting at 60% HRR in the first week and increasing by 5% every three weeks, reaching 80% HRR by the twelfth week.

The CON group underwent three weekly training sessions for twelve weeks, consisting of 20 min of aerobic activity, performed on a treadmill and rowing machine, without the WB-EMS superimposition.

### 2.4. Testing Procedures

In the baseline assessment, sample information was collected, including demographics (age, sex, education level), current or past medical conditions, pharmacological therapy, and previously performed surgical interventions.

### 2.5. Outcome Measures

The test used to assess physical fitness included measures of muscular endurance (30 s arm curl test [15], 30 s sit-to-stand test [16], and hand grip strength test [17]), cardiorespiratory fitness (6 min walking test) [18], agility and walking speed (8-foot up and go test) [19], flexibility (chair sit and reach test) [20], and hand-eye coordination (soda pop test) [21]. Quality, distance, step length, and static and dynamic balance were recorded using the Tinetti Assessment Tool [22].

Cognitive assessment was performed using the Mini-Mental State Examination (MMSE) [23], Rey’s Auditory Verbal Learning Test (RAVLT) [24], the Stroop Color and Word Interference Test (SCWT) [25], the Trail Making Test (TMT) [26], the Attentional Matrices Test (AMT) [27], the Prose Memory Test (Prose) [27], and the Frontal Assessment Battery (FAB) [28].

### 2.6. Statistical Analysis

Statistical analyses were performed using SPSS software version 23.0 (IBM, Chicago, IL, USA). Variables were tested for normal distribution using the Shapiro–Wilk test, and the significance level for statistical tests was set at 0.05. Descriptive statistics were reported as the mean and standard deviation (SD). The physical and cognitive variables followed a normal distribution; therefore, repeated measures of RM-ANOVA were conducted to assess between-group differences (ETG vs. CON, and male vs. female) and within-group differences (pre- vs. post-intervention). Additionally, the time*group interaction was calculated. The dependent variables considered for the analysis included groups (ETG and CON) and gender (male and female). The independent variables included the scores from the physical (arm curl, sit-to-stand, hand grip, 6-MWT, 8-FUG, CSR, soda pop, Tinetti) and cognitive tests (MMSE, RAVLT, SCWT, TMT, AMT, Prose, FAB). Moreover, arm curl and handgrip tests were evaluated for both limbs (right vs. left).

## 3. Results

### 3.1. Physical Performance Results

Significant differences were observed in both groups, but the interaction analysis highlighted a significantly greater effect of WB-EMS in the arm curl (*p* < 0.001), Tinetti balance and gait test (*p* = 0.012), and the 6-min walk test (*p* = 0.003).

In the sit-to-stand test, significant improvement was seen only in the ETG (*p* < 0.001).

Regarding the soda pop test (*p* < 0.001), handgrip (*p* < 0.001), flexibility (*p* < 0.001), and the 8-foot up-and-go test (*p* < 0.001), both groups showed improvements without significant differences between groups, indicating that the two training protocols produced similar effects.

The results of the physical performance tests are reported in Table 2 and Table 3.

### 3.2. Cognitive Assessment Results

Significant time effects were found for the Stroop t reaction time (*p* < 0.001), FAB (*p* < 0.001), and attention matrices (*p* < 0.001), showing improvement in both the ETG and CON groups pre- and post-intervention. In the tests performed, significant interactions were found in the RAVLT immediate (*p* < 0.001) and attention matrices (*p* = 0.017). Furthermore, differences between groups were found, in which the ETG achieved better results than the CON in regards to the RAVLT immediate (*p* < 0.001), PROSE (*p* = 0.046), and attention matrices (*p* = 0.014). Conversely, no significant results were found for the MMSE (*p* = 0.628), Stroop error (*p* = 0.188), or TMT (*p* = 0.698) for either group.

The cognitive performance results are reported in Table 4 and Table 5.

## 4. Discussion

This study aimed to assess the effect of twelve weeks of WB-EMS, combined with aerobic training (rowing machine and treadmill), on physical and cognitive performance in older adults, healthy or at risk of dementia, compared to the same training protocol without the WB-EMS.

It is well recognized that regular, long-term aerobic physical activity not only improves cardiovascular health and physical performance but also plays a crucial role in preventing dementia-related conditions. By mitigating brain volume loss, enhancing synaptic communication, and improving reaction times, aerobic exercise can slow cognitive decline in older adults [29,30].

The results of this study showed positive outcomes for motor and cognitive functions for both ETG and CON. However, the presence of interactions favoring the experimental group and significant differences in the improvement of several physical and cognitive functions confirmed the hypothesis that the application of WB-EMS enhances the effects of physical exercise in the elderly population.

Generally, scientific studies were based on strength training with WB-EMS, following the recommended protocol (rectangular stimulation at 85 Hz, 350 μs, 4 s on/4 s off). Only one previous study [31] combined low-intensity WB-EMS with aerobic exercise on a rowing machine, using the recommended protocol (rectangular stimulation at 7 Hz, 350 μs, with continuous pulse duration) [32].

Considering the positive results from the previous study, we tried to apply a higher stimulation frequency to aerobic exercise with a safe stimulation at a continuous frequency of 35 Hz. Subsequently, we compared these results to those of a control group who performed the same protocol, without the superimposition of WB-EMS, to isolate the effects of this technology. Since we fully agree with the assumption that WB-EMS is a highly safe exercise technology when used correctly [33,34], no injuries or health issues were observed during the treatment, likely due to the particular attention given to two key aspects: the use of medical WB-EMS settings, such as Miha-Bodytec technology, and the expertise and careful supervision of specialized staff in consistently managing vulnerable cohorts with appropriate care [34].

### 4.1. Physical Assessment

After 12 weeks of training, a significant improvement in almost all physical performances, compared to those noted at the beginning of the trial, were found for both groups. As expected, results in favor of the WB-EMS protocol were found for the strength tests for both upper and lower limbs, and consequently, for balance as well. An early improvement in both lower and upper limb strength could have significant potential benefits for gait and posture, helping to counteract the decline commonly observed in older adults [35].

No significant differences or time–group interactions were observed in regards to flexibility and range of motion, despite previous results assessing a significant increase in flexibility after WB-EMS exposure [36,37,38]. One possible explanation could be related to the age of the participants, as the natural decline in functional fitness with advancing age means that significant improvement often requires a longer period of exposure. Handgrip strength was positively affected by the exercise interventions in both groups, with no significant differences between groups [39,40].

In aerobic endurance tests (6-min walking test) significant differences between groups were found [41]. Continuous exercise on a rowing ergometer and treadmill not only led to significant improvements in aerobic fitness and maximal strength of the leg extensors, arms, and trunk muscles but also enhanced coordination [42]. In particular, using WB-EMS, these active movements, performed by varying joint angles and adjusting muscle length, resulted in increased spatial recruitment in the ETG group [43].

Both groups showed improvement in walking performance and balance, as measured by the Tinetti test. However, the interaction time*groups indicated that the WB-EMS protocol enhanced stability and accuracy in force production, along with the simultaneous activation of both agonist and antagonist muscles [44]. This improvement is particularly significant, as physical efficiency supports autonomy and self-sufficiency in older adults [45].

Significant improvements in movement speed and agility were observed through the execution of the 8-foot up-and-go test in both groups, which assesses the efficiency of executive cognitive functions [46]. Since movements like turning, standing up, and sitting down are more reflective of everyday activities, improvements in these areas could enhance autonomy in managing daily tasks. Conversely, reduced walking speed and slow gait are linked to a higher risk of cognitive decline and dementia in the elderly, potentially explaining the connection between sarcopenia and cognitive impairment [47].

Manual dexterity and hand–eye coordination, as assessed by the soda pop test, did not differ significantly between the ETG and CON groups, failing to confirm the neural adaptations observed in other studies following WB-EMS exposure [48].

### 4.2. Cognitive Assessment

ETG showed significant improvements in memory performances (PROSE, RAVLT immediate test), suggesting that immediate memory was more responsive to WB-EMS; this in confirmed by Xiu et al. [49], who found that higher levels of physical activity are associated with more stable cognitive function.

Conversely, no significant differences between groups were found in long-term memory (RAVLT test) over time in either the ETG or CON group. A previous study by Vázquez-Lorente (2023) [50] indicated that only high-intensity training, combined with WB-EMS, could potentially enhance long-term memory in middle-aged adults. Since previous studies have linked improvements in memory retention to elevated BDNF levels stimulated by aerobic exercise [51,52], the time*group interaction in the current study, which indicates a trend of improvement in the ETG, could postulate that a prolonged period of aerobic training, combined with WB-EMS, might also help to induce improvements in long-term memory.

Significant results regarding selective attention, cognitive flexibility, and visuospatial processing skills were found among all the participants. In the Stroop test, both groups showed improvements over time; however, the ETG showed a more significant improvement compared to the CON, suggesting that the WB-EMS intervention positively influenced the ability to inhibit automatic responses and manage conflicts between competing information, which is a crucial aspect of cognitive functioning [53]. In the attention matrices, both groups showed significant improvements, with the ETG demonstrating better results from the intervention. This indicates progress in selective attention and visuospatial processing capabilities, highlighting the effectiveness of the WB-EMS training program in enhancing the effects of physical activity [53].

In the Frontal Assessment Battery (FAB), a significant difference was observed between pre- and post-assessments in both groups, without any time*group interaction. Previous studies have indicated that both aerobic and resistance training, or a combination of the two, positively influence this cognitive function [54,55].

No significant time-effect was found in the MMSE scores and the Trail Making Test (TMT). A twelve-week period may not be sufficient to expect meaningful changes, despite findings from previous studies [5,56]. However, no deterioration in overall cognitive function was observed in this older population sample after three months.

Regarding gender differences, no significant differences between groups were observed for the cognitive variables evaluated.

## 5. Conclusions

Exploring new methods to enhance quality of life and cognitive health in older adults is crucial. WB-EMS is a time-efficient, non-invasive technology that allows for the creation of personalized exercise programs [41]. Research indicates that WB-EMS can significantly improve strength and other parameters [13] by activating both fast and slow motor units, offering notable benefits, especially for older adults [43,57].

The new proposal of combining low-frequency WB-EMS with aerobic training—which is widely recognized as the most effective form of exercise for improving physical and cognitive health in the elderly— enhanced the effects of aerobic training and represents a strength of this study. To the best of our knowledge, no study has applied WB-EMS to aerobic exercise, with continuous stimulation at a frequency of 35 Hz.

In addition to the direct benefits on the structural and functional mechanisms of the nervous system [58] in fit individuals engaged in aerobic training, the cognitive benefits of aerobic exercise may also be promoted through indirect mechanisms [59]. These mechanisms include improvements in overall health, such as reduced stress and sleep disturbances, as well as a lower prevalence of chronic conditions (e.g., inflammation and coronary artery disease), which can also influence neurocognitive function [60].

### Limitations

Due to the challenge of increasing the sample size, since the WB-EMS protocol is administered individually, a limitation of the study is the relatively limited number of participants.

Moreover, it is well known that the conversion from MCI to dementia requires a longer period of assessment and considering that the experimental protocol duration was only 12 weeks, further studies could evaluate a longer period for the WB-EMS protocol.

The study did not evaluate psychological symptoms such as depression, and considering that MCI could present such symptoms, it is likely that some of the significant results observed in this study could be related to an improvement in patient’s mood.

Furthermore, the lack of familiarity with WB-EMS [61] and the short duration of this project may have led to a lack of significant long-term cognitive changes or related adaptations from the WB-EMS application.

## Figures and Tables

**Table 1:** Sample characteristics.

Variable	Age	PASE	MAC-Q
**Sample (n = ** **61)**	71.00 ± 5.64	127.65 ± 50.34	23.24 ± 2.36
**ETG (n = 33)**	70.00 ± 6.52	131.40 ± 44.68	23.73 ± 2.61
**CON (n = 28)**	71.87 ± 4.56	123.89 ± 55.87	22.76 ± 2.00
**Male**	70.36 ± 6.53	128.16 ± 42.61	23.57 ± 2.24
**Female**	75.82 ± 5.21	127 ± 52.59	23.19 ± 2.54

PASE—Physical Activity Scale for Elderly; MAC-Q—Memory Assessment Clinics-Questionnaire.

**Table 2:** Results of physical performance for the groups. Data are reported as mean (SD).

Variable	ETG		CON		Group-Effect F_(1.59)_	Time-Effect F_(1.59)_	Interaction F_(1.59)_
	Pre	Post	Pre	Post			
**Arm curl D**	20.3(2.1)	24.9(2.4)	20.3(1.8)	22.3(1.8)	**F = 0.008** ***p* = 0.008**	**F = 157.904** ***p* < 0.001**	**F = 23.533** ***p* < 0.001**
**Arm curl ND**	21.1(2.2)	24.5(1.8)	20.8(1.8)	23.6(1.7)	F = 3.755 *p* = 0.057	**F = 77.096** ***p* < 0.001**	F = 0.681 *p* = 0.413
**Sit to stand**	14.4(1.9)	17.4(1.8)	14.8(1.7)	15.4(2)	**F = 11.758** ***p* = 0.001**	**F = 25.872** ***p* < 0.001**	**F = 8.135** ***p* = 0.006**
**Soda pop**	7.7(1)	6.4(0.5)	7.5(1.5)	6.7(1.5)	F = 0.099 *p* = 0.755	**F = 21.111** ***p* < 0.001**	F = 0.572 *p* = 0.453
**Handgrip D**	23.7(2)	25.5(2.2)	23.7(1.8)	26.2(2)	F = 0.641 *p* = 0.427	**F = 45.366** ***p* < 0.001**	F = 1.302 *p* = 0.258
**Handgrip ND**	22.1(1.5)	24.8(1.8)	21.8(1.9)	23.4(1.9)	**F = 7.766** ***p* = 0.007**	**F = 46.717** ***p* < 0.001**	F = 2.898 *p* = 0.094
**6-MWT**	469(71.1)	553(67.2)	435.1(79.4)	465.4(71.9)	**F = 13.695** ***p* < 0.001**	**F = 44.361** ***p* < 0.001**	**F = 9.843** ***p* = 0.003**
**8-FUG**	7.4(1.8)	6.3(1.6)	8(2.1)	7.1(2)	F = 2.722 *p* = 0.104	**F = 14.416** ***p* < 0.001**	F = 0.330 *p* = 0.568
**Tinetti**	26(1.3)	27.6(0.6)	26.2(1.3)	27.3(1.3)	F = 0.020 *p* = 0.887	**F = 100.054** ***p* < 0.001**	**F = 6.682** ***p* = 0.012**
**CSR D**	0.5(1.9)	1.5(1.9)	0.1(1.4)	1.4(1.6)	F = 0.813 *p* = 0.371	**F = 13.955** ***p* < 0.001**	F = 0.090 *p* = 0.765
**CSR ND**	0.6(1.9)	1.8(1.5)	0.3(1.5)	2(1.8)	F = 0.008 *p* = 0.927	**F = 29.133** ***p* < 0.001**	F = 0.718 *p* = 0.400

ETG: experimental training group; CON: control group; D: dominant; ND: non-dominant; 6-MWT: 6-min walk test; 8-FUG: 8-foot up and go; CSR: chair sit and reach test; bold: indicates significant values obtained by the RM-ANOVA.

**Table 3:** Results of physical performance by gender. Data are reported as mean (SD).

Variable	Male		Female		Group-Effect F_(1.59)_	Time-Effect F_(1.59)_	Interaction F_(1.59)_
	Pre	Post	Pre	Post			
**Arm curl D**	20.3(2.3)	23.9(2.5)	20.4(1.6)	23.6(2.5)	F = 0.010 *p* = 0.920	**F = 121.756** ***p* < 0.001**	F = 0.311 *p* = 0.579
**Arm curl ND**	21.4(2.2)	24.1(1.7)	20.6(1.8)	24.1(1.9)	F = 1.350 *p* = 0.250	**F = 79.003** ***p* < 0.001**	F = 1.066 *p* = 0.306
**Sit to stand**	14.7(1.6)	16.4(2)	15.5(2)	16.6(2.3)	F = 1.430 *p* = 0.237	**F = 25.900** ***p* < 0.001**	F = 1.547 *p* = 0.219
**Soda pop**	7.6(1.4)	6.6(1)	7.6(1.1)	6.5(1.1)	F = 0.387 *p* = 0.536	**F = 21.601** ***p* < 0.001**	F = 0.075 *p* = 0.785
**Handgrip D**	23.8(1.5)	26.4(2.1)	23.7(2.2)	25.2(2)	F = 2.241 *p* = 0.140	**F = 46.215** ***p* < 0.001**	F = 3.224 *p* = 0.078
**Handgrip ND**	22.1(1.8)	24.2(1.9)	21.8(1.5)	24.1(2)	F = 0.377 *p* = 0.541	**F = 46.705** ***p* < 0.001**	F =0.161 *p* =0.690
**6-MWT**	474.5(59.4)	523.3(82.9)	433(85.9)	502.8(80.7)	F = 0.277 *p* = 0.601	**F = 41.963** ***p* < 0.001**	F = 0.133 *p* = 0.254
**8-FUG**	7.7(2.2)	6.7(1.6)	7.5(1.7)	6.7(2)	F = 3.053 *p* = 0.086	**F = 95.154** ***p* < 0.001**	F = 0.389 *p* = 0.535
**Tinetti**	26(1.2)	27.4(1)	26.2(1.4)	27.5(1)	F = 0.089 *p* = 0.767	**F = 14.909** ***p* < 0.001**	F = 0.234 *p* = 0.630
**CSR D**	0.1(1.1)	1.4(1.7)	0.6(2)	1.5(1.7)	F = 1.491 *p* = 0.227	**F = 14.079** ***p* < 0.001**	F = 0.613 *p* = 0.437
**CSR ND**	0.3(1.6)	1.6(1.3)	0.6(1.9)	2.1(1.8)	F = 1.622 *p* = 0.208	**F = 28.425** ***p* < 0.001**	F = 0.195 *p* = 0.660

D: dominant; ND: non-dominant; 6-MWT: 6-min walk test; 8-FUG: 8-foot up and go; CSR: chair sit and reach test; bold: indicates significant values obtained by the RM-ANOVA.

**Table 4:** Results of cognitive performance for both groups: data are reported as mean (SD).

Variable	ETG		CON		Group-Effect F_(1.59)_	Time-Effect F_(1.59)_	Interaction F_(1.59)_
	Pre	Post	Pre	Post			
**MMSE**	27.3(1.5)	27.4(1.5)	27.1(1.4)	27.2(1.9)	F = 0.491 *p* = 0.486	F = 0.238 *p* = 0.628	F = 0.010 *p* = 0.921
**RAVLT Imm**	39.2(4.5)	44.9(3.7)	39.2(3.7)	37.8(4.6)	**F = 17.515** ***p* < 0.001**	**F = 11.477** ***p* = 0.001**	**F = 31.852** ***p* < 0.001**
**RAVLT diff**	9.9(3.4)	12(3.5)	10(3)	10.8(3.7)	F = 0.548 *p* = 0.462	**F = 16.114** ***p* < 0.001**	F = 3.583 *p* = 0.063
**Stroop t**	15.2(1.7)	13.2(4.1)	14.4(2.2)	13.2(2.6)	F = 0.524 *p* = 0.472	**F = 14.982** ***p <* 0.001**	F = 0.849 *p* = 0.361
**Stroop err**	0.4(2.4)	−1(4.5)	0.6(1.2)	0.7(2.3)	F = 3.038 *p* = 0.087	F = 1.778 *p* = 0.188	F = 2.166 *p* = 0.146
**TMT**	19(10.2)	18.4(9.8)	18.6(8.5)	18.4(8.2)	F = 0.012 *p* = 0.912	F = 0.152 *p* = 0.698	F = 0.054 *p* = 0.818
**FAB**	17.4(1.2)	18.4(0.8)	17.5(1.7)	18.1(1)	F = 0.135 *p* = 0.715	**F = 14.416** ***p* < 0.001**	F = 0.574 *p* = 0.451
**Prose**	24.3(1.8)	25.1(3.3)	23.7(1.4)	23.7(3.2)	**F = 4.141** ***p* = 0.046**	F = 0.933 *p* = 0.338	F = 0.852 *p* = 0.360
**Attention Matrices**	43.6(2.7)	49.4(6.6)	43(2.4)	45.5(4.3)	**F = 6.352** ***p* = 0.014**	**F = 36.538** ***p* < 0.001**	**F = 6.018** ***p* = 0.017**

ETG: experimental training group; CON: control group; RAVLT = Rey’s Auditory Verbal Learning Test; Imm = immediate; del = delayed; t = time; err = error; TMT = Trail Making Test; bold: indicates significant values obtained by the RM-ANOVA.

**Table 5:** Results of cognitive performance by gender: data are reported as mean (SD).

Variable	Male		Female		Group-Effect F_(1.59)_	Time-Effect F_(1.59)_	Interaction F_(1.59)_
	Pre	Post	Pre	Post			
**MMSE**	27.1(1.6)	27.4(1.6)	27.3(1.3)	27.2(1.8)	F = 0.015 *p* = 0.904	F = 0.256 *p* = 0.615	F = 1.332 *p* = 0.253
**RAVLT Imm**	39.9(4.1)	41.2(4.8)	38.5(4.1)	42.1(6.1)	F = 0.043 *p* = 0.837	**F = 9.902** ***p* = 0.003**	F = 2.227 *p* = 0.141
**RAVLT diff**	9.9(3.5)	11.4(3.3)	10(3)	11.5(3.6)	F =0.011 *p* = 0.915	**F = 16.516** ***p* < 0.001**	F = 0.016 *p* = 0.898
**Stroop t**	14.5(2.1)	12.9(3)	15.1(1.8)	13.5(3.9)	F = 1.054 *p* = 0.309	**F = 15.441** ***p* < 0.001**	F = 0.009 *p* = 0.924
**Stroop err**	0.4(1)	−0.6(5.1)	0.7(2.5)	0.2(1.6)	F = 1.029 *p* = 0.315	F = 2.087 *p* = 0.154	F = 0.283 *p* = 0.597
**TMT**	20.9(8.1)	19.2(8.5)	16.9(10.2)	17.6(9.6)	F = 1.907 *p* = 0.173	F = 0.185 *p* = 0.668	F = 1.040 *p* = 0.312
**FAB**	17.5(1.5)	18.2(0.9)	17.3(1.3)	18.3(0.9)	F = 0.031 *p* = 0.860	**F = 14.834** ***p* < 0.001**	F = 0.168 *p* = 0.684
**Prose**	23.9(1.7)	23.8(2.9)	24.1(1.6)	25.1(3.6)	F = 2.477 *p* = 0.121	F = 1.064 *p* = 0.306	F = 2.022 *p* = 0.160
**Attention Matrices**	43.1(2.7)	48.3(6)	43.5(2.5)	47(5.9)	F = 0.269 *p* = 0.606	**F = 36.741** ***p* < 0.001**	F = 1.447 *p* = 0.234

RAVLT = Rey’s Auditory Verbal Learning Test; Imm = immediate; del = delayed; t = time; err = error; TMT = Trail Making Test; bold: indicates significant values obtained by the RM-ANOVA.

## Data Availability

Data will be made available on request.

## Supplementary Materials

The following supporting information can be downloaded at: https://www.mdpi.com/article/10.3390/jfmk9040246/s1, File S1: WB-EMS device.
