# Supplementary material for: Enhancing Physical and Cognitive Efficiency in Elderly Individuals at Risk for Dementia Using Whole-Body Electrostimulation: A Randomized Controlled Trial
Source: J Funct Morphol Kinesiol. 2024 Nov 23;9(4):246. doi: 10.3390/jfmk9040246 (PMC11586937; doi:10.3390/jfmk9040246)
Supplement: Supplementary file 1 [file jfmk-09-00246-s001.zip › jfmk-3303787-supplementary.pdf]

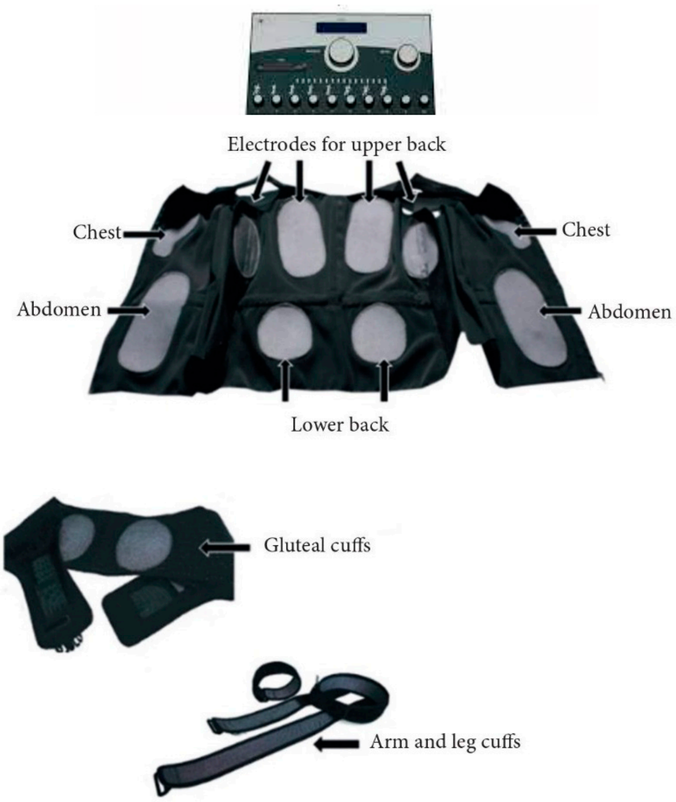

Whole-body electrical muscle stimulation (WB-EMS) is defined as "the simultaneous application of electrical stimuli through at least six current channels or the engagement of all major muscle groups, with an effective current pulse to trigger muscle adaptations." WB-EMS can simultaneously activate up to 14-18 regions across 8-12 different muscle groups over an area of 2800 cm^2, combining global electrical stimulation with functional movements. In WB-EMS, the frequency of electrical impulses, measured in Hertz (Hz), plays a crucial role in determining the type and intensity of muscle contraction. It has a range from 0 to 100 Hz.
